# Evaluation of the potential adverse effects associated with calcium carbonate precipitate during continuous venovenous hemofiltration

**DOI:** 10.1186/cc10972

**Published:** 2012-03-20

**Authors:** J McKee, B Brooks, J Daller, J Gass, D Pantaleone, P Zieske

**Affiliations:** 1 Baxter, Round Lake, IL, USA

## Introduction

This study evaluated the potential adverse effects associated with exposure to calcium carbonate precipitate during continuous venovenous hemofiltration (CVVH). The clinical use of Accusol 35 Solution (Accusol 35) has been associated with occasional formation of calcium carbonate precipitate in the tubing set during therapy.

## Methods

Fourteen mongrel dogs were anesthetized, instrumented, and received CVVH with the test (*n = *6) or negative control article (*n = *8) for 6 hours. The test article was Accusol 35 with induced precipitate formation prior to CVVH. The test article contained visible particles and subvisible particles 36× higher than the maximum concentration specified in the European Pharmacopoeia (EP). The negative control article was Accusol 35 containing no visible particles and subvisible particles within EP specification. One-half of the dogs in the negative control article group received a central venous injection of Sephadex G-50 beads (10 mg/kg) following CVVH as a positive control. Select cardiovascular (CV) parameters were monitored continuously or were calculated at predetermined times. Arterial samples were obtained at predetermined times for analysis of blood gases and electrolytes. Samples of the test and negative control articles were obtained hourly during CVVH for determination of pH and subvisible particles. Dogs were euthanized and lung tissue samples were examined histologically.

## Results

All CV parameters remained stable and no differences were observed between the test and negative control articles. Sephadex beads caused an increase (*P *< 0.01) in mean pulmonary arterial pressure due solely to a similar increase (*P *< 0.01) in pulmonary vascular resistance. No differences in blood gases or electrolytes were observed between the test and negative control articles. Sephadex beads caused a decrease (*P *> 0.05) in arterial blood PO_2 _and an increase (*P *> 0.05) in arterial blood PCO_2_. No differences in lung histology were observed between the test and negative control articles. The lungs from all dogs given Sephadex beads contained multiple intravascular particles in large-caliber blood vessels.

## Conclusion

CVVH performed on anesthetized dogs for 6 hours using Accusol 35 containing visible and subvisible particles 36× higher than the maximum concentration specified in the EP resulted in no adverse effects on CV parameters, blood gases and electrolytes, and lung histology as compared with Accusol 35 containing no visible particles and subvisible particles that were within EP specification.

